# Effectiveness and Safety of Compound Chinese Medicine plus Routine Western Medicine in In-Stent Restenosis: A Meta-Analysis and Systematic Review

**DOI:** 10.1155/2018/6207524

**Published:** 2018-07-11

**Authors:** Lu Liu, Jing Liu, Qun Gao, Yang Wu, Jinjin Lu, Jie Wan, Yan Li, Xiaoyun Cui, Kun Zhou, Wenhao Jia, Yanchao Huang, Wenbai Qu, Qian Lin

**Affiliations:** ^1^Beijing University of Chinese Medicine, North 3rd Ring East Road, Beijing 100029, China; ^2^Department of Cardiology, Dongfang Hospital, Beijing University of Chinese Medicine, Fanggu Road, Beijing 100078, China; ^3^Intensive Care Unit, Dongfang Hospital, Beijing University of Chinese Medicine, Fanggu Road, Beijing 100078, China; ^4^Scientific Research Division, Dongfang Hospital, Beijing University of Chinese Medicine, Fanggu Road, Beijing 100078, China

## Abstract

**Objective:**

To examine the effects and safety of oral compound Chinese medicine (CCM) plus routine western medicine (RWM) in in-stent restenosis (ISR).

**Methods:**

Various electronic databases (CBM, CNKI, VIP, Wanfang, PubMed, EMBASE, and Cochrane Library) were searched until April 2017. The quality of the included studies was evaluated, and meta-analyses were performed using RevMan5.3 and STATA 12.0 software. Moreover, funnel plot and Egger's publication bias plots were analysed to identify publication bias and adverse reactions were reported. A sensitive analysis was carried out according to the quality score.

**Results:**

In all, 40 RCTs involving 4536 patients were selected for this review. The pooled estimates of three studies showed that the benefit to the number of ISRs (NoR) was more substantial for CCM plus RWM than for RWM alone (RR 0.24, 95% CI 0.10 to 0.57, *P* = 0.001; *I*^2^ = 0%, *P* = 0.81). The rate of ISR was significantly lower for CCM plus RWM than for the same RWM alone (RR 0.44, 95% CI 0.37 to 0.53, *P* < 0.00001; *I*^2^ = 0%, *P* = 0.95). CCM plus RWM benefitted the rate of ISR when a CM placebo plus RWM was used as the control intervention (RR 0.34, 95% CI 0.20 to 0.57, *P* < 0.0001; *I*^2^ = 0%, *P* = 0.95). The difference of adverse reactions was not significant. For secondary outcomes, the CCM plus RWM group did not reduce the rates of revascularization and cardiac death, but it did reduce the rate of recurrent angina over the results observed in the RWM alone group. In addition, funnel plot and Egger's publication bias plot indicated that there was publication bias. The association between the use of CCM plus RWM and RWM alone remained significant after the sensitivity analysis excluding studies with low quality score (quality score ⩽ 4) with a pooled RR of 0.41 (95% CI, 0.34–0.50).

**Conclusion:**

Oral CCM plus RWM clearly benefitted patients with percutaneous coronary intervention (PCI) because it prevented and treated ISR better than was observed for either RWM alone or a CM placebo plus RWM.

## 1. Introduction

Currently, percutaneous coronary intervention (PCI) is widely used around the world to treat coronary artery disease (CAD) and has significantly reduced mortality in patients with acute coronary syndromes [1]. However, the incidence rate of in-stent restenosis (ISR) is still approximately 10%, even accounting for the introduction of drug-eluting stents (DES) [2], and reaches as high as 40%–50% in patients with multivessel involvement [3]. ISR therefore inflicts a heavy burden on both the lives of patients with CAD and the economy and remains a challenge to the implantation of PCI and CAD prognoses.

In traditional Chinese medicine (TCM), the typical symptoms of ISR, such as chest pain and chest tightness, are referred to as “Xiongbi” [4]. TCM presents mature theories and is supported by abundant clinical experience for treating “Xiongbi”. In recent years, the efficacy and safety of TCM in ISR has been widely studied with remarkable results. Studies have shown that TCM not only clearly alleviates the typical symptoms of ISR but also improves its long-term prognosis, suggesting that TCM may lead to promising applications for the treatment of ISR.

Previous reviews focused on this issue were published in 2008 [5], 2012 [6], and 2014 [7]. The first two of these studies were systematic reviews (SRs), and the latter was a meta-analysis. They explored the effects of many types of PCI and included studies with small sample sizes (fewer than 30 cases) and that explored a single TCM herb. In addition, a presearch showed that, in the 3 years since 2014, more studies have supported the efficacy and safety of oral compound Chinese medicines (CCMs) in ISR. In this SR, we applied more rigorous inclusion and exclusion criteria and aimed to explore whether oral CCM plus routine western medicine (RWM) is effective and safe for treating ISR after stent implantation. We use CCM because it has wider applications in clinical practice and conforms better to TCM theory.

## 2. Methods

This SR was performed according to the Preferred Reporting Items for Systematic Reviews and Meta-Analyses (PRISMA) Statement [8] and the Cochrane Handbook [9] Systematic review and is registered at PROSPERO with the registration number CRD42017075368 (https://www.crd.york.ac.uk/PROSPERO).

### 2.1. Search Strategy

A comprehensive search of 7 medical databases, including the PubMed, EMBASE, Cochrane Library, China National Knowledge Infrastructure (CNKI), Chinese Scientific Journals (VIP), Wanfang, and Chinese Biomedical (CBM) databases, was conducted through April 2017 without a language restriction. We performed the search using individually or combined Mesh terms for all fields relating to the patients (coronary heart disease and coronary artery disease) and interventions (traditional Chinese medicine, Chinese herbal medicine, compound Chinese medicine, Chinese prepared medicine, Chinese herbal formula, and Chinese herbs) of interest. All titles/subjects related to the outcome (in-stent restenosis) were searched. When searching Chinese databases, the above terms were searched in Chinese.

### 2.2. Inclusion Criteria

Only randomized controlled trials (RCTs) were included. We focused on trials with participants diagnosed with major angiographic criteria-documented [10] CAD who were eligible for stent implantation (with either bare metal stents or drug-eluting stents) regardless of their gender, age, disease course, comorbidity, and ethnic origin. The baseline characteristics of each study were consistent. The interventional treatment was any oral CCM plus RWM administered for at least 1 month regardless of dosage. The control group was treated with the same RWM or with a CM placebo plus the same RWM. “CCM” included Chinese prepared medicines or Chinese herbal formulas. The compositions of the CCM were detailed. Our primary outcomes were restenosis occurring within at least 6 months of follow-up. Coronary in-stent restenosis is classically defined as the angiographic detection of a recurrent stenosis with a diameter greater than 50% at the stent segment or the 5 mm segments adjacent to it (in-segment restenosis). The drop-out rate for coronary angiography (CA) review was required to be lower than 20%. Each included study was required to report the primary outcome. Secondary outcomes could be major adverse cardiac events (MACE), including recurrent angina, myocardial infarction, revascularization, and cardiac death. Only secondary outcomes that improved in equal to or more than 50% of cases were considered responses. In addition, adverse reactions were considered as a primary safety outcome. When there were several follow-up points, only the last one was considered.

### 2.3. Exclusion Criteria

Studies with a small sample study (less than 30 cases), duplicate reports, and pilot studies were excluded. Any trial that failed to satisfy the inclusion criteria or for which required data were unavailable was excluded.

### 2.4. Data Extraction

Two reviewers (Jinjin Lu and Yan Li) independently extracted the data using a standardized extraction form. Disagreements were resolved by census or consultation with a third reviewer (Wenhao Jia). The following items were extracted: study name, year of publication, sample size, details of the trial design (i.e., randomization, allocation concealment, and blinding), eligibility criteria, general characteristics of patients, details of intervention and control therapies, details related to outcomes, details related to drop-outs, and other information that may help detect bias.

### 2.5. Quality Assessment

Two reviewers (Xiaoyun Cui and Kun Zhou) independently assessed the methodological quality of the included studies using the Cochrane Collaboration's tool [11], which is a domain-based evaluation tool used to generate a “risk of bias” table for each study. Any disagreement was resolved by consensus or consultation with a third reviewer (Yanchao Huang). The domains used for assessment were sequence generation (selection bias), allocation concealment (selection bias), blinding of participants and personnel (performance bias), blinding of outcome assessment (detection bias), incomplete outcome data (attrition bias), selective outcome reporting (reporting bias), and other potential sources of bias (e.g., early termination, contamination, and conflict of interest). Because it is difficult to blind participants in studies of Chinese herbal medicines, performance bias was likely present in all trials. With regard to the objective outcomes adopted in this SR, such as ISR and MACE, performance bias and detection bias might not be as important, and we therefore summarized these trials as low-risk. In addition, with regard to selective outcomes, this domain was ranked as “low-risk” unless the outcomes were critical to our issue.

### 2.6. Data Analysis

Data synthesis and analysis were performed with RevMan software 5.3 and STATA 12.0 software (StataCorp LP, College Station, TX) [9]. Dichotomous data were measured as relative risk (RR), while continuous data were measured as the mean difference (MD), both with corresponding 95% confidence intervals (CIs). Heterogeneity across trials was measured with the Cochran *Q* test and is presented as *I*^2^ statistics. Only trials with *I*^2^ lower than 85% were used for the meta-analysis, and the characteristics of the included trials were similar. A fixed effect model was used if *I*^2^ was lower than 25%. Otherwise, a random effect model was applied under the assumption that any heterogeneity was readily explainable. Furthermore, funnel plot and Egger's test were performed to detect heterogeneity and publication bias, respectively, if sufficient sources were available. A 2-tailed *P* value less than 0.05 was considered significant. A sensitivity analysis was conducted to assess the stability of the results.

## 3. Results

### 3.1. Study Identification

**Figure 1:**
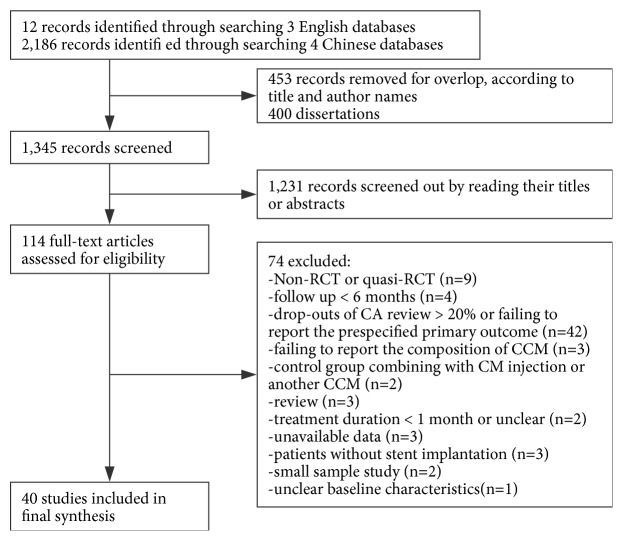
Study flow diagram.

All eligible studies were screened and identified (Figure 1). A total of 2198 records were retrieved. Of these, full-text evaluations were conducted on 114 studies. In all, 74 of these 114 studies were excluded for the following reasons: non-RCT or quasi-RCT (*n* = 9); follow-up < 6 months (*n* = 4); drop-outs from CA review > 20% or failure to report the prespecified primary outcome (*n* = 42); failure to report the composition of the CCM (*n* = 3); control group consisted of a CM injection or another CCM (*n* = 2); review (*n* = 3); treatment duration < 1 month or unclear (*n* = 2); unavailable data (*n* = 3); patients without stent implantation (*n* = 3); small study sample size (*n* = 2); and unclear baseline characteristics (*n* = 1). Finally, a total of 40 RCTs with a total of 4536 patients were included in this SR [12–51].

### 3.2. Characteristics of Included RCTs

All studies were conducted in China from 2010 to 2017. The average age of the patients ranged from 52.93 to 69.27 years old. Most trials had more males than females. The diagnostic criteria for CAD were mainly based on CA criteria or the Nomenclature and Diagnostic Criteria for Ischemic Heart Disease (World Health Organization, WHO) [52]. All patients successfully underwent stent implantation. The distributions of baseline characteristics were basically the same in each group. Four trials [25, 26, 36, 49] were double-blinded, and, in three trials, the control group used a CM placebo plus RWM [19, 25, 26]. The remaining trials were designed to compare a CCM plus RWM group to a group treated with same RWM alone. Of the 40 studies, 20 used a decoction, 1 used an oral liquid, 2 used granules, 4 used pills, 12 used capsules, and 1 used tablets. RWM mainly consisted of aspirin, Clopidogrel, angiotensin-converting inhibitor (ACEI)/angiotensin II receptor blocker (ARB), beta-blockers, and statins. The dosage and types of RWM were prescribed according to the recommendations of Chinese Society of Cardiology Guideline [10]. Overall, 33 kinds of CCM were used, and the treatment courses ranged from 3 to 12 months with a follow-up time of 6 to 18 months. For outcomes, ISR was assessed by computed tomography angiography (CTA) or CA in all trials; adverse reactions caused by CCMs were reported in 11 studies but were significant in only 2 studies [26, 38]; MACE were reported in 12 studies and included recurrent angina (*n* = 12), myocardial infarction (*n* = 3), revascularization (*n* = 2), and cardiac death (*n* = 5) (Tables 1 and 2).

### 3.3. Methodological Quality of Included RCTs

**Figure 2:**
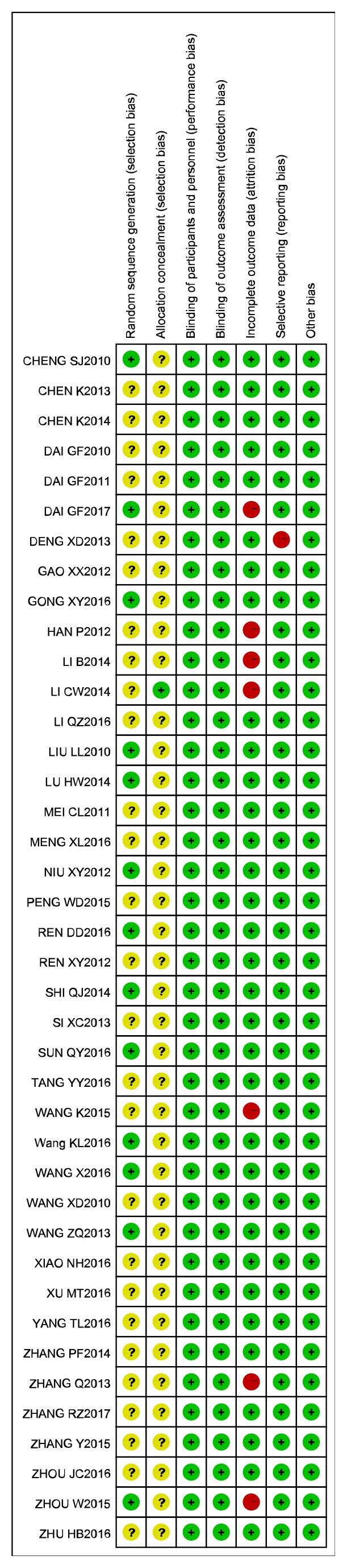
Summary of risk of bias in included studies.

Of the 40 included studies, 13 were randomized by random number tables or SPSS software. The other 27 studies used the phrase “randomly allocating” but did not describe the method of randomization. Only 1 trial reported allocation concealment as “by sealed, opaque envelopes”. Participants or outcome assessors were blinded in only 4 trials. All studies reported the primary outcome and clearly described the data collection methods, and we therefore believed that they were free from selective reporting bias. According to the criteria we prespecified, as shown in the Quality Assessment section, we judged the performance bias and detection bias of these studies to be low-risk. In conclusion, the overall methodological quality of the 40 trials was rated as low-risk. The details of this analysis are shown in Figure 2. The mean score for the RCTs included in this analysis was 5.15 (Table 3).

### 3.4. Primary Outcomes

#### 3.4.1. In-Stent Restenosis

**Figure 3:**
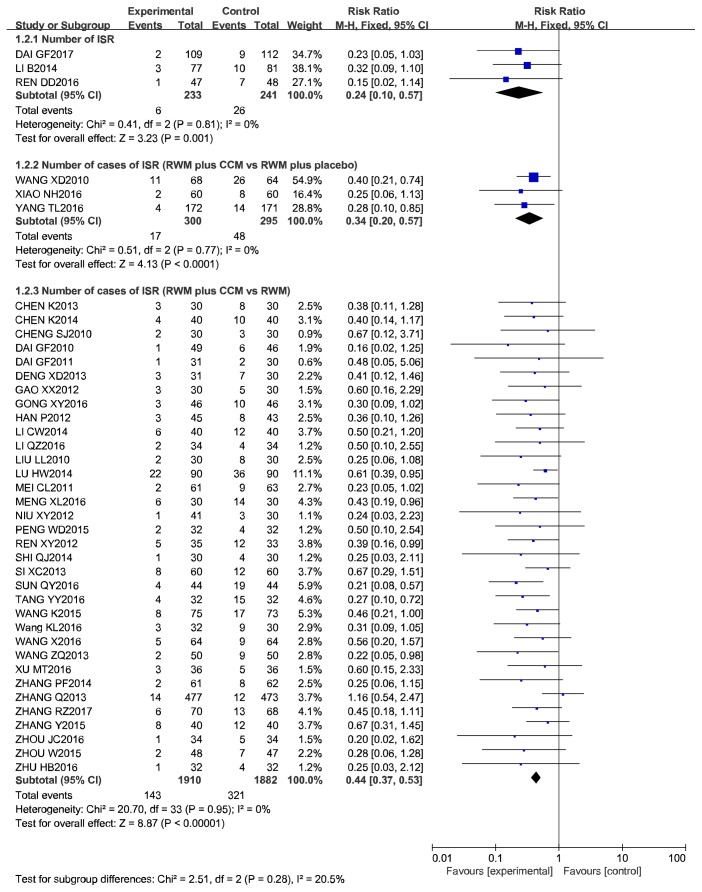
Meta-analysis of the effectiveness of CCM plus RWM in IRS.

**Figure 4:**
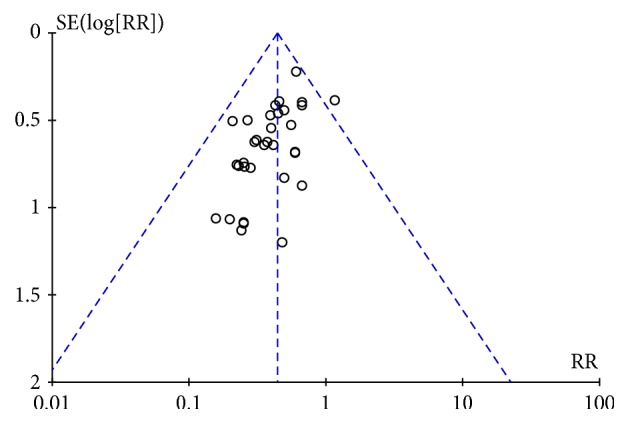
Funnel Plot of CCM plus RWM versus RWM.

**Figure 5:**
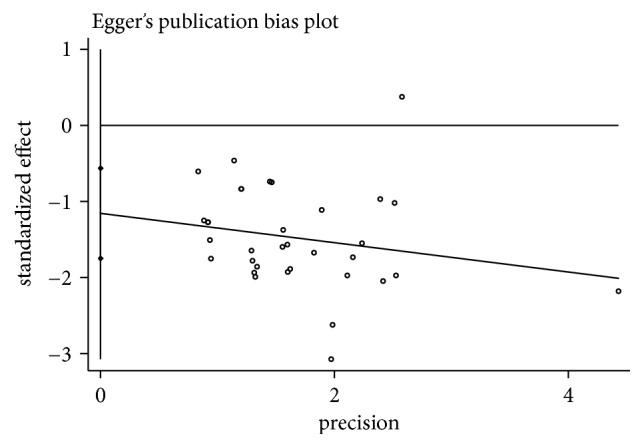
Egger's publication bias plot for CCM plus RWM vs RWM in cases of ISR.

Three studies provided data for the number of ISR (NoR), and the pooled estimates showed that, in patients with CAD, NoR was lower for CCM plus RWM than for RWM alone (RR 0.24, 95% CI 0.10 to 0.57, *P* = 0.001; *I*^2^ = 0%, *P* = 0.81) after 6 months of follow-up. In all, 34 studies provided data on the number of cases of ISR that had a follow-up time of 6 to 18 months. The rate of ISR was obviously lower in the CCM plus RWM group than in the group treated with the same RWM alone (RR 0.44, 95% CI 0.37 to 0.53, *P* < 0.00001; *I*^2^ = 0%, *P* = 0.95). A meta-analysis of an additional 3 studies also showed that CCM plus RWM exerted a beneficial effect on the rate of ISR when CM placebo plus RWM was used as the control intervention with a 6- to 12-month follow-up (RR 0.34, 95% CI 0.20 to 0.57, *P* < 0.0001; *I*^2^ = 0%, *P* = 0.95). In addition, we evaluated these 34 studies to detect publication bias. The funnel plot was asymmetrical, indicating publication bias. Because funnel plot is used as a qualitative method to detect the publication bias of an article, we also used Egger's method to detect publication bias, and the results again implied the presence of publication bias (*P* = 0.00 < 0.05). The details of these analyses are shown in Figures 3–5 and Table 4.

#### 3.4.2. Subgroup Analysis

**Figure 6:**
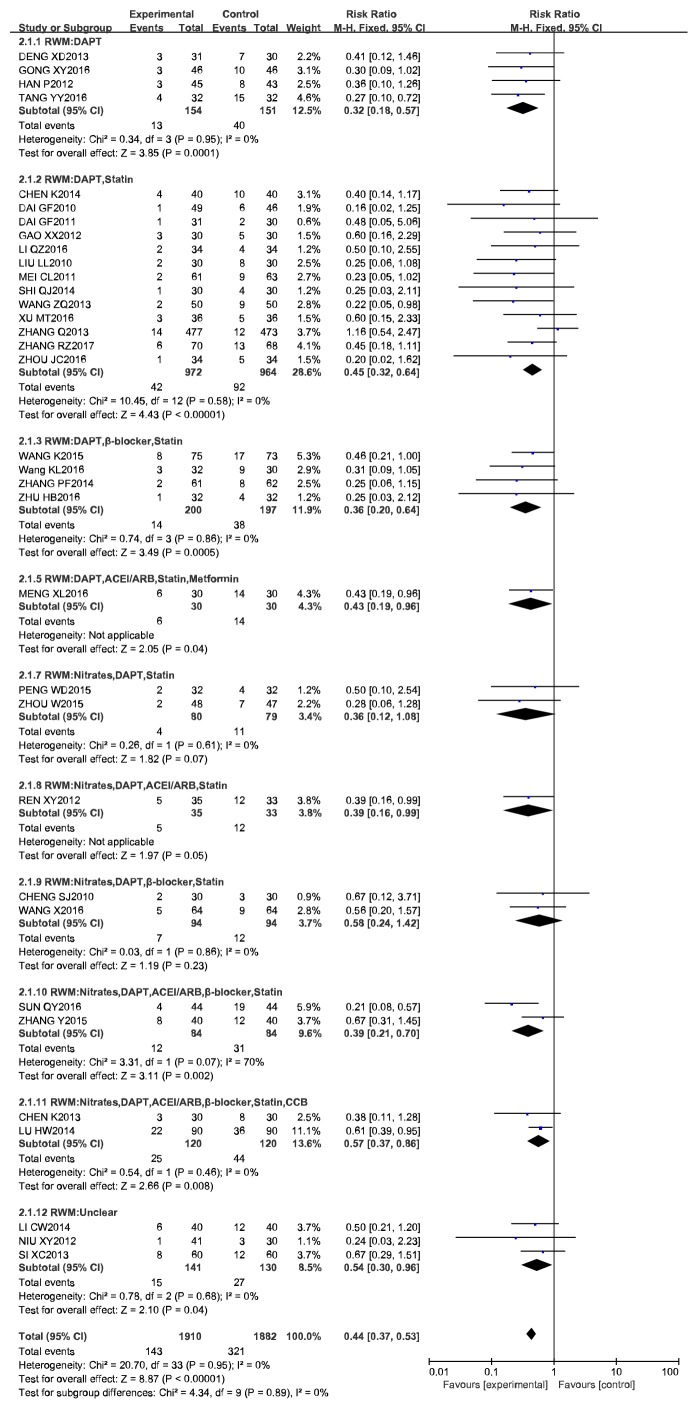
Meta-analysis of CCM plus RWM versus RWM in cases of ISR.

Different types of Western medicine were used among the 34 studies, and we therefore performed a subgroup analysis. In 25 studies, a better benefit was exerted on ISR by CCM plus RWM (DAPT, DAPT + Statin, DAPT + *β*-blocker + Statin, Nitrates + DAPT + ACEI/ARB + *β*-blocker + Statin, Nitrates + DAPT + ACEI/ARB + *β*-blocker + Statin + CCB) than the RWM alone. In 4 studies, there was no significant difference between the CCM plus RWM (Nitrates + DAPT + Statin, Nitrates + DAPT + ACEI/ARB + *β*-blocker + Statin) group and the RWM group (Table 5 and Figure 6).

**Figure 7:**
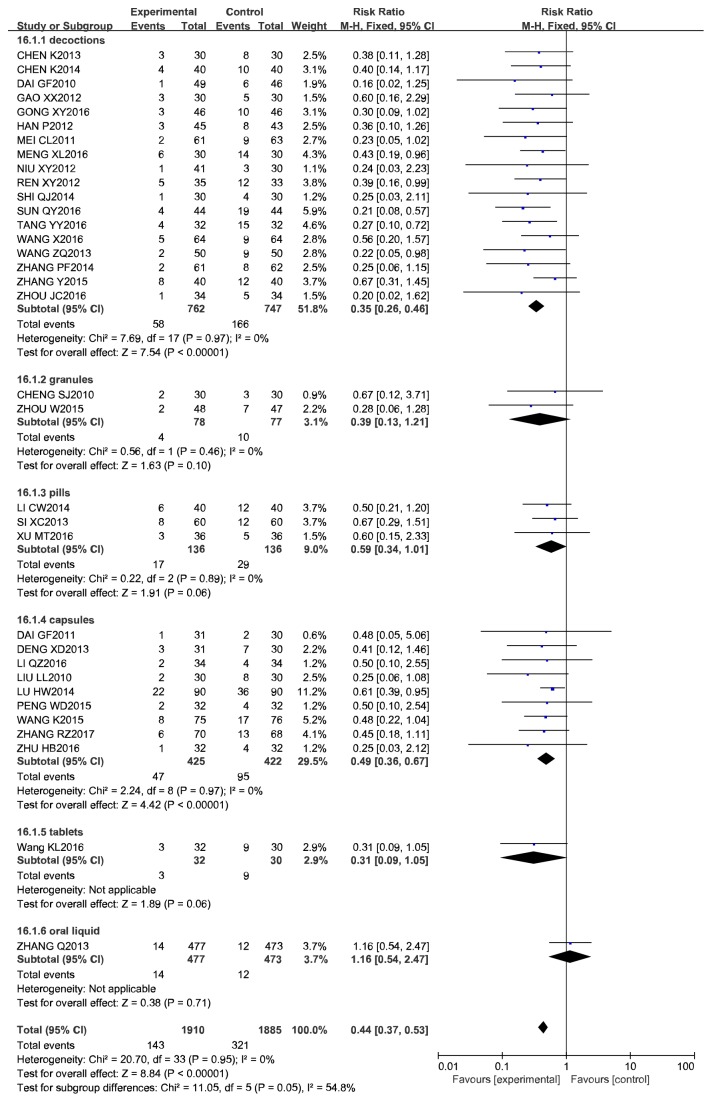
Meta-analysis of different CCM plus RWM versus RWM in cases of ISR.

Difference in the dosage forms of drugs (e.g., decoction, granules, pills, capsules, and tablets) might impact results, and we therefore carried out a subgroup analysis. A pooled estimate of cases of ISR showed that there was a significant difference between the decoction plus RWM and RWM alone groups in 18 studies (RR 0.28, 95% CI = 0.20 to 0.38, *P* < 0.00001; *I*^2^ = 0%, *P* = 0.97). No additional benefit was exerted in ISR in the granules plus RWM group than in the RWM alone group (RR 0.39, 95% CI 0.13 to 1.21, *P* = 0.10; *I*^2^ = 0%, *P* = 0.46). A meta-analysis of another 3 studies also showed that there was no significant difference between the pills plus RWM group and the RWM alone group (RR 0.59, 95% CI 0.34 to 1.01, *P* = 0.60; *I*^2^ = 0%, *P* = 0.89). There were significantly fewer cases of ISR in the capsules plus RWM group than in the group treated with the same RWM alone (RR 0.49, 95% CI 0.36 to 0.67, *P* < 0.00001; *I*^2^ = 0%, *P* = 0.97). The details of these analyses are shown in Table 6 and Figure 7.

**Figure 8:**
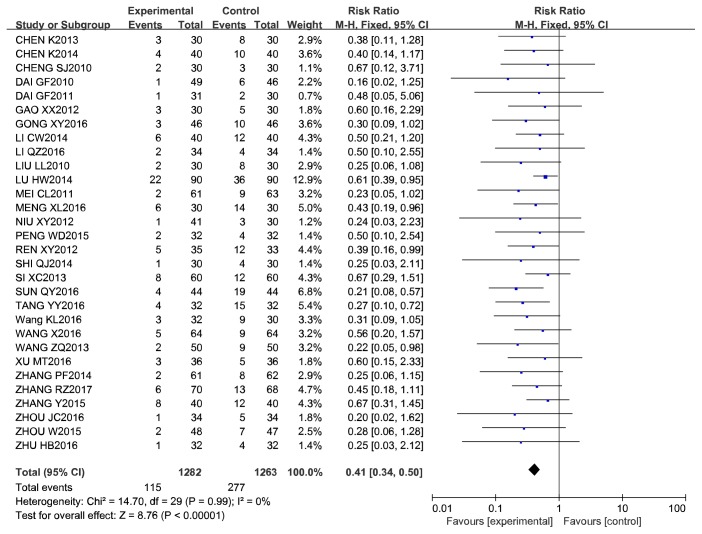
Sensitivity analysis excluding studies with low quality score of CCM plus RWM versus RWM in cases of ISR.

The association between the use of CCM plus RWM and RWM alone remained significant after the sensitivity analysis excluding studies with low quality score (quality score  ⩽ 4) with a pooled RR of 0.41 (95% CI, 0.34–0.50). (Figure 8).

#### 3.4.3. Adverse Reactions

**Figure 9:**
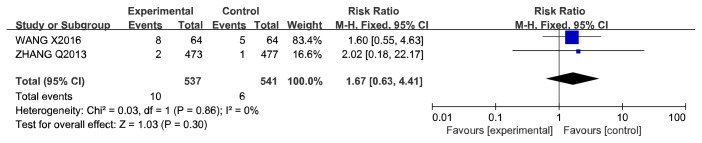
Meta-analysis of adverse reactions in the CCM plus RWM versus RWM groups.

Only 2 studies reported the details of adverse reactions related to CCM, including hepatic dysfunction, gingival bleeding, diarrhoea, stomach discomfort, flushing, dizziness, and headache. More adverse reactions were reported in patients treated with a Huoxue yiqi decoction and a Shenshao decoction, but most of these reactions were not severe and disappeared without special treatment. There was no significant difference in adverse reactions between the CCM plus RWM and RWM alone groups (Table 7 and Figure 9).

### 3.5. Secondary Outcomes

#### 3.5.1. Major Adverse Cardiac Events (Table 8)

**Figure 10:**
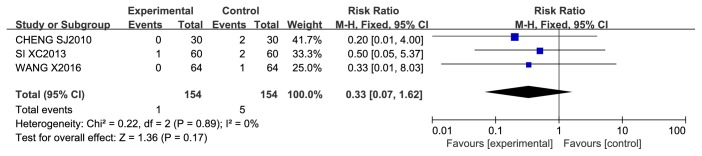
Meta-analysis of revascularization in CCM plus RWM versus RWM alone.

*Revascularization.* Revascularization was reported in 3 studies, in which 308 patients were treated with 3 different CCMs, including Anxin granules, Shexiang baoxin pills, and a Huoxue yiqi tongmai decoction. After 6–18 months of follow-up, the rate of revascularization was not significantly different between the CCM plus RWM and the RWM alone groups (RR 0.33, 95% CI 0.07 to 1.62, *P* = 0.17; *I*^2^ = 0%, *P* = 0.89) (Figure 10).

**Figure 11:**
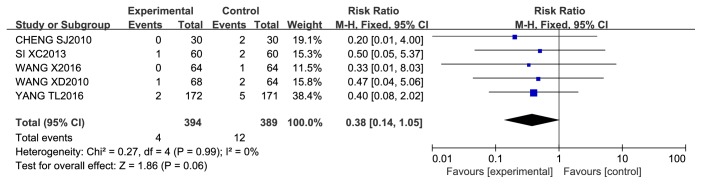
Meta-analysis of myocardial infarction in CCM plus RWM versus RWM.

*Myocardial Infarction.* Myocardial infarction (MI) was reported in 5 studies, in which 783 patients were treated with 4 different CCMs, including Anxin granules, Shexiang baoxin pills, Tongxinluo capsules, and a Huoxue yiqi tongmai decoction. During approximately 6–18 months of follow-up, no significant difference was found between the CCM plus RWM group and the RWM alone group (RR 0.33, 95% CI 0.07 to 1.62, *P* = 0.17; *I*^2^ = 0%, *P* = 0.89). A meta-analysis of the other 2 studies also showed that there was no significant difference between the CCM plus RWM group and the CM placebo plus RWM group (RR 0.42, 95% CI 0.11 to 1.60, *P* = 0.20; *I*^2^ = 0%, *P* = 0.91) after 6 months of follow-up (Figure 11).

**Figure 12:**
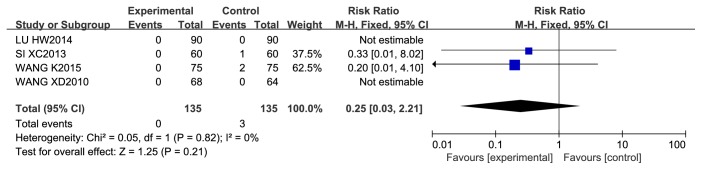
Meta-analysis of cardiac mortality in CCM plus RWM versus RWM.

*Cardiac Mortality.* In 5 studies of 4 kinds of CCM, 461 patients were included in the treatment group, and 460 patients were included in the control group. Cardiac death was recorded as a measure of effect during a 12–18-month follow-up period. No cardiac deaths occurred in any of the groups in three of the studies or in the experimental group in the other 2 studies. A pooled estimate of cardiac deaths showed that there was no significant difference between the CCM plus RWM and RWM alone groups in the latter two studies (RR 0.25, 95% CI = 0.03 to 2.21; *P* = 0.21; *I*^2^ = 0%, *P* = 0.82) (Figure 12).

**Figure 13:**
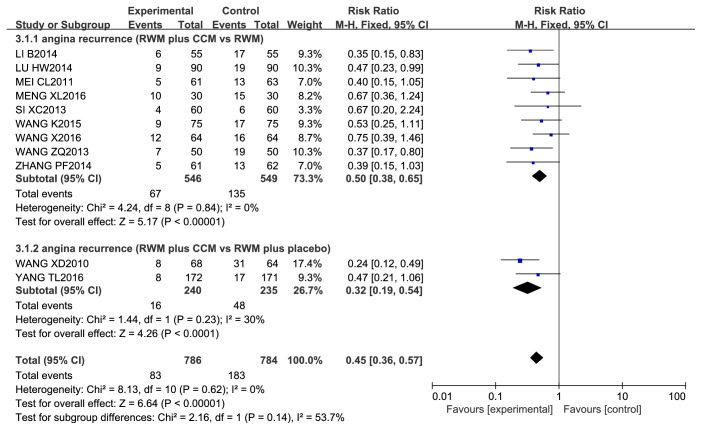
Meta-analysis of recurrent angina in CCM plus RWM versus RWM.

*Recurrent Angina.* Recurrent angina was reported in 11 studies involving 1570 patients. During a 6–18-month follow-up period, the rate of recurrent angina was significantly lower in the CCM plus RWM group than in the groups treated with the same RWM alone (RR 0.50, 95% CI 0.38 to 0.65, *P* < 0.00001; *I*^2^ = 0%, *P* = 0.84) or with a CM placebo plus the same RWM (RR 0.32, 95% CI 0.19 to 0.54, *P* < 0.00001; *I*^2^ = 30%, *P* = 0.23) (Figure 13).

*Angiographic Measurements.* Follow-up angiography performed in patients with ISR at 6–12 months after the index PCI in 7 studies that evaluated 4 Chinese Herbal Medicines (CHMs) in 1917 patients. Baseline information showed that the mean minimal luminal diameter (MLD) before and immediately after the index PCI and the gain in luminal diameter following stent placement were comparable between groups.

MLD is defined as the smallest diameter in the treated lesion area [53] and was reported in 5 studies (Table 9). MLD was significantly better in the CCM plus RWM group than in the CM placebo plus RWM when patients were treated with Tongxinluo capsules (MD_WANG XD2010_ 1.27 mm, 95% CI 1.01 to 1.53 mm; MD_YANG TL2016_ = 0.35 mm, 95% CI 0.27 to 0.43 mm) and in the CCM plus RWM group than in the group treated with the same RWM alone when a Huoxue yiqi tongmai decoction was evaluated (MD_WANG X2016_ 0.42 mm, 95% CI 0.35 to 0.49 mm). There was no significant difference between the groups in the studies evaluating Shenshao oral lotion (MD_ZHANG Q2016_ = 0.02 mm, 95% CI −0.02 to 0.06 mm). The Tongxinluo capsule plus RWM did not improve MLD when patients were compared to those treated with the same RWM alone (MD_DENG XD2013_ = −1.61 mm, 95% CI −1.93 to −1.29 mm).

The primary angiographic endpoint was late lumen loss (LLL) after 6 to 12 months. LLL was evaluated by determining the difference between the minimum lumen diameter after the procedure and at follow-up using quantitative coronary angiography [54]. LLL was measured in 4 studies of 3 CHMs after 6 and 12 months of follow-up (Table 10). There was a significant difference between the CHM plus RWM group and the control group treated with the same RWM alone when the CHM being tested was Tongxinluo capsules (MD_LU HW2014_ = −0.36 mm, 95% CI −0.42 to −0.30 mm), a Xinmai futong decoction (MD_ZHANG PF2014_ = −0.3 mm, 95% CI −0.41 to −0.19 mm) and a Shenshao oral lotion (MD_ZHANG Q2013_ = −0.05 mm, 95% CI −0.09 to −0.01 mm). Finally, LLL was lower in the RWM plus Tongxinluo capsule group than in the group treated with the same RWM plus placebo (MD_YANGN TL2016_ = −0.24 mm, 95% CI −0.28 to −0.20 mm).

In this SR, 40 studies involving 4536 CAD patients who underwent stent implantation were identified. All studies were RCTs. Approximately 75% of the studies were performed in the last five years. A domain-based evaluation showed that the mean score of the included RCTs was 5.15. Of the included studies, three were designed to compare a CCM plus RWM versus a CM placebo plus the same RWM, and 37 studies were designed to compare a CCM plus RWM versus the same RWM alone.

In this SR, all studies assessed ISR using CTA or CA. A three subgroup meta-analysis of 33 CCM plus RWM groups showed that they produced an absolute decrease in the ISR rate. Given the low risk of bias (demonstrated by analyses of methodological quality and publication bias), the following robust conclusion can be drawn: CCM plus RWM reduces ISR. Furthermore, there was no significant difference in adverse reactions between the CCM plus RWM and RWM alone groups in 2 of the studies, potentially indicating that CCM plus RWM is a safe treatment. However, further observations are needed. An Egger's linear regression test was performed to statistically assess funnel plot asymmetry and publication bias. Substantially more studies in the literature have positive than negative results, and this produced so-called publication bias.

With regard to secondary outcomes, we concluded that CCM plus RWM did not exert a benefit against revascularization, MI and cardiac mortality during a 6–18-month follow-up period after stent implantation. This finding may be attributed to the limited number of patients and the short follow-up times reported in the studies. However, 11 studies explored CCMs plus RWMs, and recurrent angina was significantly reduced by this treatment during the 6–18-month follow-up period. This may indicate that treatment with a CCM plus RWM exerts a better effect on symptom improvement. Some studies have concluded that a major determinant of the restenosis rate is the final MLD after all interventions [55]. MLD can be evaluated as a continuous end point from 6 to 9 months after intervention and is precisely associated with stable long-term results in the treated coronary segment [56]. In addition, LLL is monotonically correlated with the probability of restenosis and, when evaluated, is a more efficient estimate of restenosis progress in this era of lower binary restenosis rates [54]. Hence, MLD and LLL are two major determinants of the restenosis rate. In this SR, 7 studies that evaluated 4 CHMs reported MLD and LLL. Although pooled results were not available for either MLD or LLL because there was high heterogeneity (*I*^2^ = 97% or *I*^2^ = 99%), in most of the studies, CCM plus RWM exerted significant beneficial effects.

Specifically, Tongxinluo capsules appeared to markedly reduce the rates of ISR, recurrent angina, and LLL and improve MLD. Qiwei sanxiong decoction [37] appeared to markedly reduce the rates of ISR and recurrent angina. Yiqi huoxue huayu decoction [24], Yiqi huayu decoction [43], Yixin tongmai decoction [22], and Yiqi tongluo huatan decoction [16] each provided significant benefits by reducing the rate of ISR. Qishen yiqi droplets [35] exerted significant benefits by reducing recurrent angina. Most of the above-described CCMs are meant to supplement Qi and activate the blood. Their main ingredients are Huangqi (astragalus), Renshen (Panax), Shaoyao (peony), Danshen (salvia), and Chuanxiong (tetramethylpyrazine).

The pathogenesis of restenosis is not yet fully understood. However, it is generally accepted that when a stent causes injury to a coronary artery, it triggers a series of inflammatory reactions that result in the migration and proliferation of vascular smooth muscle cells (SMCs) within the vessel lumen and neointimal hyperplasia. In TCM, CAD is thought to be a result of “Qi deficiency and Blood Stasis”. The severity of Blood Stasis syndrome is significantly correlated with the complexity of coronary lesions and the degree of stenosis and is an important factor affecting the occurrence of restenosis after PCI [57]. Many studies have demonstrated that Astragalus [58], salvia [59], tetramethylpyrazine [60], and Chuangxingol and paeoniflorin [61], which are main ingredients in TCM, play effective anti-inflammatory roles and inhibit SMC proliferation and migration. This may be the mechanism by which the above-mentioned CCMs prevent and treat restenosis.

Ideally, RCTs should adhere to known research design standards. In our examination of these studies, we did not have access to enough details related to these characteristics, although the baselines were comparable between comparison groups in the included studies. Details about randomization methodology were also lacking. In the 40 studies we reviewed, 11 reported that they implemented randomization using a random number table or a computer random number generator, such as SPSS software, and only 1 mentioned using sealed, opaque envelope concealment. Participants or outcome assessors were blinded in only 4 of the trials. In addition, studies that involve therapeutic trials should also report the rates of adverse reactions regardless of whether or not they occur. Reporting of adverse reactions is very important for evaluating the safety of interventional measures even though there is no guarantee that the adverse reactions were related to the interventional measure. Subgroup analyses showed that different types of Western medicines and drug forms affected outcomes. Furthermore, most of the 40 studies failed to mention the type of stent that was deployed (i.e., whether it was a bare metal stent or a drug-eluting stent). Therefore, the difference in the curative effects observed between the CCM plus RWM and control groups is uncertain, and there is bias in our results. In our review, only 11 of the 40 included trials reported adverse reactions, rendering it difficult to systematically evaluate the safety of the evaluated CCMs in ISR. Future research on these topics will help to clarify the effectiveness and safety of CCMs in ISR.

## 4. Conclusions

Based on the results of this SR, we conclude combining RWM with CCMs may provide moderate efficacy in preventing ISR following PCI with stent placement. This is despite the fact that our investigation revealed the potential presence of bias in the identified studies. CCMs used to supplement Qi and activate the blood are suggested for preventing and treating restenosis. In addition, additional experimental studies should explore the mechanisms by which the main ingredients in CCM act to supplement Qi or activating the blood to prevent and treat restenosis. Future rigorously designed RCTs that explore CCM plus RWM therapies aimed at preventing post-PCI ISR should adhere to established design standards to overcome the limitations presented in this review. In particular, they should ensure adequate concealment of allocation and blinding of primary outcomes assessors.

## Figures and Tables

**Table 1:** Baseline characteristics of included patients.

Author, Year	No. of patients (EG/CG)	Age, mean (SD/range), EG/CG, year	Gender, M/F, N.	Co-morbidities, N. EG/CG	Lesion site, EG/CG	Lesion number, EG/CG	Stent number, EG/CG	CAD diagnostic guidelines
DAI GF 2017	117 (57/60)	63.5 (9.82) 65.07/(8.99)	E: 36/21 C39/21	HTN: 40/41 DM: 33/34 Dyslipidaemia: 49/51	LMCA: 4/3 LAD: 12/13 LCX: 9/10 RAC: 10/11	DBL: 15/16 TBL: 4/5 MBL: 3/2	109/112	CA

ZHANG RZ 2017	138 (70/68)	59.4 (8.5)/57.3 (6.4)	E: 41/29 C: 40/28	HTN: 14/13 DM: 10/9 Hyperlipidemia: 12/10	NR	NR	NR	2007: Guideline for Percutaneous Coronary Intervention (CSC)

WANG KL 2016	62 (32/30)	58.93 (5.52)/60.43 (5.39)	E: 22/10 C: 16/14	HTN: 25/23 DM: 8/9 Hyperlipidemia: 11/13	NR	SBL: 9/8 TBL: 17/15 TBL: 6/8	1.83 ± 0.58/1.98 ± 0.70	CA

GONG XY 2016	92 (46/46)	57.8 (1.9)/57.5 (2.1)	E: 21/25 C: 20/26	NR	NR	NR	NR	Nomenclature and diagnostic criteria of Ischemic heart disease (WHO)

TANG YY 2016	64 (32/32)	52.32 (4.12)/53.53 (4.55)	E: 20/12 C: 22/10	HTN: 20/19 DM: 19/17 Hyperlipidemia: 25/24	NR	NR	NR	CA

ZHOU JC 2016	68 (34/34)	NR	NR	NR	NR	NR	NR	Nomenclature and diagnostic criteria of Ischemic heart disease (WHO)

ZHU HB 2016	64 (32/32)	61.5 (5.5)/60.5 (5.8)	E: 19/13 C: 18/14	NR	NR	NR	NR	Nomenclature and diagnostic criteria of Ischemic heart disease (WHO)

XIAO NH 2016	120 (60/60)	63.15 (9.45)/63.83 (9.50)	E: 40/20 C: 39/21	HTN: 44/43 DM: 14/18	NR	SBL: 18/19 TBL: 20/19 TBL: 22/22	137/150	CA
REN DD 2016	60 (30/30)	55.4 (9.78)/55.76 (9.25)	E: 16/14 C: 18/12	HTN: 14/13 DM: 4/5	NR	SBL: 16/17 TBL: 10/9 TBL: 4/4	48/47	CA

LI QZ 2016	68 (34/34)	60.2 (4.3)/61.3 (5.2)	E: 20/14 C: 19/15	NR	NR	NR	NR	Nomenclature and diagnostic criteria of Ischemic heart disease (WHO)

SUN QY 2016	88 (44/44)	63.1 (3.2)/62.5 (2.6)	E: 29/15 C: 28/16	HTN: 19/18 DM: 10/9 Hyperlipidemia: 13/14	NR	NR	NR	CA

XU MT 2016	72 (36/36)	58.2 (4.5)/60.5 (10.5)	E: 21/15 C: 20/15	NR	NR	NR	NR	Nomenclature and diagnostic criteria of Ischemic heart disease (WHO)

MENG XL 2016	60 (30/30)	60 (46~72)/61.5 (49~73)	E: 16/14 C: 13/17	HTN: 12/10 DM: 5/5 Arrhythmia: 8/9	NR	NR	NR	*⟪*Internal medicine*⟫*

YANG TL 2016	343 (172/171)	57.93 (9.77)/58.63 (10.27)	E: 137/35 C: 134/37	HTN: 105/102 DM: 172/171	LMCA: 6/7 LAD: 117/111 LCX: 101/92 RAC: 77/88	SBL: 66/69 DBL: 86/77 MBL: 23/25	301/298	CA

WANG X 2016	128 (64/64)	65.8 (4.4)/65.3 (4.2)	E: 49/15 C: 48/16	NR	NR	SBL: 26/25 DBL: 23/22 MBL: 16/17	NR	*⟪*Practice of internal medicine*⟫*
ZHOU W 2015	100 (50/50)	69.08 (7.46)/69.46 (7.35)	E: 28/22 C: 27/23	HTN: 23/24 DM: 9/10 Hyperlipidemia: 20/19	NR	NR	NR	2009: ACC/AHA guidelines for the management of patients with ST-elevation myocardial infarction and ACC/AHA/SCAI guidelines on percutaneous coronary intervention.

PENG WD 2015	64 (32/32)	61.5 (5.5)/60.5 (5.8)	E: 19/13 C: 18/14	NR	NR	NR	NR	Nomenclature and diagnostic criteria of Ischemic heart disease (WHO)

ZHANG Y 2015	80 (40/40)	53.2 (5.4)/51.9 (5.3)	E: 21/19 C: 22/18	NR	NR	NR	NR	CA

WANG K 2015	148 (75/73)	57.6 (10.3)/56.5 (9.1)	E: 59/16 C: 56/19	HTN: 32/34 DM: 23/28 Hyperlipidemia: 55/45	NR	NR	NR	CA

LU HW 2014	180 (90/90)	60.2 (6.9)/61.8 (7.2)	E: 48/42 C: 46/44	HTN: 59/53 DM: 23/25 Hyperlipidemia: 13/17	NR	SBL: 36/32 DBL: 33/40 TBL: 21/18	132/127	Nomenclature and diagnostic criteria of Ischemic heart disease (WHO), 2009: Guideline for Percutaneous Coronary Intervention (CSC)

ZHANG PF 2014	123 (61/62)	57.44 (8.07)/60.64 (8.91)	E: 39/22 C: 38/24	NR	NR	NR	NR	CA

SHI QJ 2014	60 (30/30)	60.3 (6.2)/62.8 (6.2)	E: 20/10 C: 18/12	NR	NR	NR	NR	Nomenclature and diagnostic criteria of Ischemic heart disease (WHO)

CHEN K 2014	80 (40/40)	56.2 (5.2)/57.9 (5.3)	E: 19/21 C: 20/20	NR	NR	NR	NR	CA
LI B 2014	102 (51/51)	61.2 (5.8)/60.6 (5.8)	E: 38/17 C: 36/19	HTN: 22/24 DM: 10/11 Hyperlipidemia: 30/25	NR	NR	77/81	CA

LI CW 2014	83 (43/40)	53.14 (9.45)/54.19 (10.7)	E: 27/16 C: 22/18	NR	NR	NR	NR	2007: Guideline for Percutaneous Coronary Intervention (CSC)

WANG ZQ 2013	100 (50/50)	56.92 (12.21)/58.90 (13.19)	E:28/22 C:27/23	HTN: 14/16 DM: 8/9 Hyperlipidemia: 12/11	NR	SBL: 12/10	NR	Nomenclature and diagnostic criteria of Ischemic heart disease (WHO)

ZHANG Q 2013	1123 (509/514)	55.6 ± 7.9/55.5 ± 7.8	E: 438/71 C: 441/73	HTN: 274/29 DM: 139/127 Stroke: 3/26	NR	NR	NR	Nomenclature and diagnostic criteria of Ischemic heart disease (WHO)

DENG XD 2013	62 (31/30)	59.0 (42~76)/59.5 (41~78)	E: 21/10 C: 20/10	NR	LAD: 20/18 LCX: 2/1 RAC: 9/11	NR	NR	Nomenclature and diagnostic criteria of Ischemic heart disease (WHO)

CHEN K 2013	60 (30/30)	55.2 (5.2)/54.9 (5.3)	E: 16/14 C: 15/15	NR	NR	NR	NR	CA

SI XC 2013	120 (60/60)	NR	NR	NR	NR	NR	NR	Nomenclature and diagnostic criteria of Ischemic heart disease (WHO)

HAN P 2012	102 (54/48)	NR	NR	NR	NR	NR	NR	CA

REN XY 2012	68 (35/33)	56.2 (5.3)/55.9 (5.8)	E: 18/17 C: 17/16	NR	NR	NR	NR	Nomenclature and diagnostic criteria of Ischemic heart disease (WHO)

NIU XY 2012	71 (41/30)	60.5 (6.50)/61.8 (6.1)	E: 25/16 C: 18/12	NR	NR	NR	NR	Nomenclature and diagnostic criteria of Ischemic heart disease (WHO)

GAO XX 2012	60 (30/30)	56.5 (5.3)/55.9 (5.8)	E: 16/14 C: 15/15	HTN: 9/11 DM: 11/10	NR	NR	NR	CA
DAI GF 2011	61 (31/30)	61.3 (7.23)/63.5 (6.1)	E: 19/12 C: 17/13	NR	NR	NR	NR	Nomenclature and diagnostic criteria of Ischemic heart disease (WHO) 1979

MEI CL 2011	124 (61/63)	56.3 (5.6)/58.3 (6.1)	E: 35/26 C: 36/27	DM: 16/17 HTN: 20/23	NR	NR	NR	Nomenclature and diagnostic criteria of Ischemic heart disease (WHO)

WANG XD 2010	132 (68/64)	65.2 (9.9)/65.1 (10.4)	T: 43/25 C: 45/19	HTN: 59/61 DM: 68/64 Hyperlipidemia: 52/49	LAD: 36/34 LCX: 33/26 RAC: 26/28	SBL: 32/26 DBL: 34/35 TBL: 2/3	131/126	CA

CHENG SJ 2010	60 (30/30)	57.9 (5.2)/58.1 (3.9)	T: 20/10 C: 22/8	HTN: 16/13 DM: 6/9 hyperlipidemia: 12/11	NR	SBL: 19/23 MBL: 11/7	NR	Nomenclature and diagnostic criteria of Ischemic heart disease (WHO)

DAI GF 2010	94 (49/45)	61.2 (6.3)/63.8 (5.9)	T: 38/21 C: 26/20	NR	NR	NR	NR	Nomenclature and diagnostic criteria of Ischemic heart disease (WHO)

LIU LL 2010	60 (30/30)	63.57 (50~75)/62.8 (50~68)	T: 19/11 C: 18/12	HTN: 22/19 DM: 12/14 Hyperlipidemia: 5/6	NR	NR	NR	2007: Guideline for Percutaneous Coronary Intervention (CSC)

EG = experimental group; CG = control group; SD = standard deviation; M/F = male/female; N = number of subjects; CAD = coronary artery disease; HTN = hypertension; DM = diabetes mellitus; LMCA = left main coronary artery; LAD = left anterior descending coronary; LCX = left circumflex; RAC = right coronary artery; DBL = double branch Lesions; TBL = three branch Lesions; MBL = multiple branch lesions; CA = coronary angiography; NR = not reported; CSC = Chinese Society of Cardiology; WHO = World Health Organization; SBL: Single branch Lesion; ACC = American College of Cardiology; AHA = American Heart Association; SCAI, Society for Cardiovascular Angiography and Interventions.

**Table 2:** Descriptive summary of treatment parameters of included studies.

Author, year	Intervention			Treatment course (mos)	Follow-up (mos)	outcomes
	CG		EG			
DAI GF 2017	RWM:	Clopidogrel P.O. 75 mg qd, Aspirin P.O. 0.1g qd, Atorvastatin P.O. 20 mg qd.	RWM plus Tongmai decoction P.O. 100 ml bid	6	6	ISR, Response rate of ECG

ZHANG RZ 2017	RWM:	Clopidogrel P.O. 75 mg qd, Aspirin P.O. 0.1 g qd, Atorvastatin P.O. 20 mg qd.	RWM plus Huoxue Tongluo capsule P.O. 4 pills tid	6	6	ISR, Response rate of ECG

WANG KL 2016	RWM:	Clopidogrel P.O. 75 mg qd, Aspirin P.O. 0.1 g qd, Metoprolol tartrate P.O. 12.5 mg bid, Atorvastatin P.O. 10 mg qd.	RWM plus Huxinkang tablets P.O. 10 pills tid	6	6	ISR, Response rate of ECG

GONG XY 2016	RWM:	Clopidogrel P.O. 75 mg qd, Aspirin P.O. 100 mg qd.	RWM plus Huoxue tongmai decoction P.O. 150 ml bid	6	6	ISR

TANG YY 2016	RWM:	Clopidogrel P.O. 75 mg qd, Aspirin P.O. 0.1 g qd.	RWM plus Yiqi tongluo huatan decoction P.O. 1 dose bid	6	6	ISR

ZHOU JC 2016	RWM:	Clopidogrel P.O. 75 mg qd, Aspirin P.O. 0.1g qd, Atorvastatin P.O. 20 mg qd.	RWM plus Qiwei sanxiong decoction P.O. 1/2 dose bid	6	6	ISR

ZHU HB 2016	RWM:	Clopidogrel P.O. 75 mg qd, Aspirin P.O. 0.5 g qd, Metoprolol tartrate P.O. 75 mg qd, Atorvastatin P.O. 10 mg qd.	RWM plus Mailuotong capsule P.O. 0.84 g tid.	6	6	ISR, Response rate of ECG

XIAO NH 2016	RWM:	Aspirin, Clopidogrel, ACEI/ARB, Beta blocker, Rosuvastatin P.O. 10 mg qd,	RWM plus Xinxuetong capsule P.O. 2 pills tid.	6	6	ISR
	Plus placebo P.O. 2 pills tid.					

REN DD 2016	RWM:	Clopidogrel P.O. 75 mg qd, Aspirin P.O. 0.1 g qd, Metoprolol tartrate P.O. 25 mg bid, Rosuvastatin P.O. 10 mg qd.	RWM plus Huoxue tongmai decoction P.O. 200 ml bid.	6	6	ISR
LI QZ 2016	RWM:	Clopidogrel P.O.75 mg qd, Aspirin P.O. 100 mg qd, Atorvastatin P.O. 10 mg qd.	RWM plus Guanxin tongluo capsule P.O. 1.6 g tid	6	6	ISR, Response rate of ECG

SUN QY 2016	RWM:	Aspirin P.O. 100 mg qd, Benazepril hydrochloride P.O. 10 mg qd, Clopidogrel P.O. 75 mg qd, Isosorbide mononitrate P.O. 25 mg tid, Metoprolol tartrate P.O. 12.5 mg bid, Simvastatin P.O. 10 mg qd.	RWM plus Yixin tongmai decoction P.O. 1/2 dose bid.	6	6	ISR

XU MT 2016	RWM:	Clopidogrel P.O. 75 mg qd, Aspirin P.O. 0.1 g qd, Atorvastatin P.O. 10 mg qd	RWM plus Guanxin suhe wan P.O. 30 g tid.	6	6	ISR

MENG XL 2016	RWM:	Clopidogrel P.O. 75 mg qd, Aspirin P.O. 0.1 g qd, Atorvastatin P.O. 20 mg qd, Valsartan P.O. 80 mg qd, Metformin P.O. 0.5 g bid.	RWM plus Yiqi Huoxue Jiedu decoction P.O. 300 ml bid.	6	6	ISR, Angina recurrence

YANG TL 2016	RWM:	Aspirin P.O. 100 mg qd, Clopidogrel P.O. 75 mg qd, pioglitazone 30 mg/d, ACEI/ARB, Beta blocker, Statins.	RWM plus Tongxinluo capsule P.O. 1.04 g tid.	12	12	ISR, CD, Angina recurrence, MI, MLD, LLL
	plus placebo P.O. 1.04 g tid.					

WANG X 2016	RWM:	Aspirin P.O.100 mg qd, Clopidogrel P.O. 75 mg qd, Atorvastatin P.O. 20 mg qd, Isosorbide mononitrate P.O. 40 mg qd, Metoprolol P.O. 23.75 mg qd.	RWM plus Huoxue yiqi tongmai decoction 100 ml bid.	6	6	ISR, MI, PCI, MLD, Angina recurrence

ZHOU W 2015	RWM:	Clopidogrel P.O. 75 mg qd, Aspirin P.O.0.1 g qd, Simvastatin P.O. 20 mg qd, Glyceryl trinitrate P.O. 0.5 mg prn.	RWM plus Longzhi dispensing granules P.O. 36 g tid.	6	6	ISR
PENG WD 2015	RWM:	Clopidogrel P.O. 75 mg qd, Aspirin P.O. 0.5 g qd, Atorvastatin P.O. 10 mg qd, Metoprolol tartrate P.O. 75 mg qd.	RWM plus Mailuo shutong capsule P.O. 0.84 g tid	6	6	ISR, Response rate of ECG

ZHANG Y 2015	RWM:	Aspirin, Clopidogrel, Nitrates, ACEI, Beta blocker, Statins.	RWM plus No. 1 Xintong prescription P.O. 100 ml bid.	6	6	ISR

WANG K 2015	RWM:	Clopidogrel P.O. 75 mg qd, Aspirin P.O. 100 mg qd, Atorvastatin P.O. 40 mg qd, Metoprolo P.O. 25 mg qd.	RWM plus Xuefu zhuyu capsule P.O. 3 pills tid.	12	12	ISR, CD, Angina recurrence

LU HW 2014	RWM:	Aspirin, Clopidogrel, Nitrates, ACEI, Beta blocker, Statins, CCB.	RWM plus Tongxinluo capsule P.O. 3 pills tid.	12	12	ISR, CD, MI, LLL, Angina recurrence

ZHANG PF 2014	RWM:	Clopidogrel P.O. 75 mg qd, Atorvastatin P.O. 20 mg qd, Metoprolol tartrate P.O. 12.5 mg bid.	RWM plus Xinmai futong decoction P.O. 1/2 dose bid.	6	6	ISR, LLL, Angina recurrence

SHI QJ 2014	RWM:	Clopidogrel P.O. 75 mg qd, Aspirin P.O. 0.1 g qd, Atorvastatin P.O. 20 mg qd.	RWM plus Yiqi huayu jiedu decoction P.O. 1/2 dose bid.	6	6	ISR, Response rate of ECG

CHEN K 2014	RWM:	Aspirin, Clopidogrel, Statins, ACEI, Beta blocker, nitrates, CCB.	RWM plus No. 2 Xintong prescription P.O. 200 ml bid.	6	6	ISR

LI B 2014	RWM:	Aspirin, Clopidogrel, Nitrates, Beta blockers, Statins.	RWM plus Qishen yiqi droplets P.O. 0.5 tid.	6	6	ISR, Angina recurrence, Response rate of ECG

LI CW 2014	RWM		RWM plus Qishen yiqi droplets P.O. 0.5 tid.	9	9	ISR
WANG ZQ 2013	RWM:	Aspirin P.O.0.1 g qd, Clopidogrel P.O. 75 mg qd, Atorvastatin P.O. 20 mg qd.	RWM plus Qiwei sanxiong decotion P.O. 150 ml bid.	6	6	ISR, Angina recurrence

ZHANG Q 2013	RWM:	Clopidogrel P.O. 75 mg qd, Aspirin P.O.0.1 g qd, Atorvastatin P.O. 20 mg qd.	RWM plus Shenshao oral lotion P.O. 10 ml tid.	12	12	ISR, MLD, LLL

DENG XD 2013	RWM:	Aspirin P.O.300 mg/d, Clopidogrel P.O. 75 mg/d.	RWM plus Tongxinluo capsule P.O. 4 pills tid	6	6	ISR, MLD

CHEN K 2013	RWM:	Aspirin, Clopidogrel, Statins, ACEI, Beta blockers, Nitrates, CCB.	RWM plus No. 3 Xintong prescription P.O. 100 ml bid.	12	12	ISR

SI XC 2013	RWM		RWM plus Shexiang baoxin wan P.O. 2 pills tid.	6	18	ISR, CD, MI, Angina recurrence, Revascularization

HAN P 2012	RWM:	Aspirin P.O. 300 mg (0–3 m),75–150 mg qd (3–6 m) Clopidogrel P.O. 75 mg qd.	RWM plus combined decoction (Gualou xiebai banxia decoction, Xuefu zhuyu decoction) P.O. 100 ml bid.	6	6	ISR

REN XY 2012	RWM:	Aspirin, Clopidogrel, nitrate, Statins, ACEI.	RWM plus Yiqi huayu decoction P.O. 100 ml bid.	6	6	ISR

NIU XY 2012	RWM		RWM plus Jingqi danshen decoction P.O. 97 g bid.	3	6	ISR Response rate of ECG

GAO XX 2012	RWM:	Aspirin, Clopidogrel, Statins.	RWM plus No. 1 Xintong prescription P.O. 100 ml bid.	6	6	ISR
DAI GF 2011	RWM:	Clopidogrel P.O. 75 mg qd, Aspirin P.O.0.1 g qd, Atorvastatin P.O. 20 mg qd.	RWM plus Tongxinluo capsule P.O.	6	6	ISR, Response rate of ECG

MEI CL 2011	RWM:	Clopidogrel P.O. 75 mg qd, Aspirin P.O.0.1 g qd, Simvastatin P.O. 20 mg qd.	RWM plus Xueyu decoction P.O. 100 ml bid.	3	6	ISR, Angina recurrence

WANG XD 2010	RWM:	Aspirin P.O. 300 mg (0-1m), 100 mg (1–12 m) qd, Clopidogrel P.O. 75 mg qd, Atorvastatin P.O. 20 mg qd.	RWM plus Tongxinluo capsule P.O. 1.14 g tid.	12	12	ISR, Angina recurrence, MI, CD, MLD
	Plus placebo P.O. 1.14g tid.					

CHENG SJ 2010	RWM:	Aspirin, Clopidogrel, Glyceryl trinitrate, Metoprolol, Atorvastatin.	RWM plus Anxin granules P.O. 3.5 g tid.	6	6	ISR, MI, PCI

DAI GF 2010	RWM:	Clopidogrel P.O. 75 mg qd, Atorvastatin P.O. 20 mg qd. Aspirin P.O. 100 mg qd,	RWM plus Yiqi huoxue tongmai decoction P.O. 250 ml bid.	12	12	ISR, Response rate of ECG

LIU LL 2010	RWM:	Clopidogrel P.O. 75 mg qd, Atorvastatin P.O. 20 mg qd. Aspirin P.O. 100 mg qd,	RWM plus Jingtian zaitong capsule P.O. 10 g tid.	6	6	ISR, Response rate of ECG

EG = experimental group; CG = control group; RWM = routine western medicine; ECG = electrocardiogram; ACEI = angiotensin-converting enzyme inhibitor; ARB = angiotensin II receptor blockage; CD = cardiac death; MI = myocardial Infarction; MLD = minimum lumen diameter; LLL = late loss of lumen; PCI = percutaneous coronary intervention; CCB = calcium channel blocker.

**(a):** 

Quality assessment criteria	DAI GF 2017	CHEN K2013	CHEN K2014	CHENG SJ2010	DAI GF 2010	DAI GF 2011	DENG XD2013	GAO XX2012	GONG XY2016	HAN P2012	LI B2014	LI CW 2014	LI QZ 2016	LIU LL 2010
Random sequence generation	+	-	-	+	-	-	-	-	+	-	-	-	-	+
Allocation concealment	-	-	-	-	-	-	-	-	-	-	-	+	-	-
Blinding of participants and personnel	+	+	+	+	+	+	+	+	+	+	+	+	+	+
Blinding of outcome assessment	+	+	+	+	+	+	+	+	+	+	+	+	+	+
Incomplete outcome data	-	+	+	+	+	+	+	+	+	-	-	-	+	+
Selective reporting	+	+	+	+	+	+	-	+	+	+	+	+	+	+
Other bias	+	+	+	+	+	+	+	+	+	+	+	+	+	+
Overall quality score (maximum = 7)	5	5	5	6	5	5	4	5	6	4	4	5	5	6

**(b):** 

Quality assessment criteria	LU HW 2014	MEI CL 2011	MENG XL2016	NIU XY2012	PENG WD2015	REN DD2016	REN XY2012	SHI QJ2014	SI XC 2013	SUN QY2016	TANG YY2016	WANG K2015	WANG KL2016
Random sequence generation	+	-	-	+	-	+	-	+	-	+	-	-	+
Allocation concealment	-	-	-	-	-	-	-	-	-	-	-	-	-
Blinding of participants and personnel	+	+	+	+	+	+	+	+	+	+	+	+	+
Blinding of outcome assessment	+	+	+	+	+	+	+	+	+	+	+	+	+
Incomplete outcome data	+	+	+	+	+	+	+	+	+	+	+	-	+
Selective reporting	+	+	+	+	+	+	+	+	+	+	+	+	+
Other bias	+	+	+	+	+	+	+	+	+	+	+	+	+
Overall quality score (maximum = 7)	6	5	5	6	5	6	5	6	5	6	5	4	6

**(c):** 

Quality assessment criteria	WANG X2016	WANG XD2010	WANG ZQ2013	XIAO NH2016	XU MT2016	YANG TL2016	ZHANG PF2014	ZHANG Q2013	ZHANG RZ2017	ZHANG Y2015	ZHOU JC2016	ZHOU W2015	ZHU HB2016
Random sequence generation	+	-	+	-	-	-	-	-	-	-	-	+	-
Allocation concealment	-	-	-	-	-	-	-	-	-	-	-	-	-
Blinding of participants and personnel	+	+	+	+	+	+	+	+	+	+	+	+	+
Blinding of outcome assessment	+	+	+	+	+	+	+	+	+	+	+	+	+
Incomplete outcome data	+	+	+	+	+	+	+	-	+	+	+	-	+
Selective reporting	+	+	+	+	+	+	+	+	+	+	+	+	+
Other bias	+	+	+	+	+	+	+	+	+	+	+	+	+
Overall quality score (maximum = 7)	6	5	6	5	5	5	5	4	5	5	5	5	5

**Table 4:** Egger's test.

Std_Eff	Coef.	Std. Err.	*t*	*P* > |*t*|	[95% Conf. Interval]	
slope	−.192833	.1590705	−1.21	0.234	−0.5168491	.1311831
bias	−1.155985	.3911951	−3.97	0.000	−1.74913	−.56284

**Table 5:** Subgroup analysis of CCM plus RWM versus RWM in cases of ISR.

RWM	Studies (*n*)	RR (95% CI)	*I* ^2^
DAPT	4	0.32 [0.18, 0.57]	0%
DAPT + Statin	13	0.45 [0.32, 0.64]	0%
DAPT + *β*-blocker + Statin	4	0.36 [0.20, 0.64]	0%
DAPT + ACEI/ARB + Statin + Metformin	1		
Nitrates + DAPT + Statin	2	0.36 [0.12, 1.08]	0%
Nitrates + DAPT + ACEI/ARB + Statin	1		
Nitrates + DAPT + *β*-blocker + Statin	2	0.58 [0.24, 1.42]	0%
Nitrates + DAPT + ACEI/ARB + *β*-blocker + Statin	2	0.39 [0.21, 0.70]	70%
Nitrates + DAPT + ACEI/ARB + *β*-blocker + Statin + CCB	2	0.57 [0.37, 0.86]	0%
Unclear	3		

**Table 6:** Subgroup analysis of CCM plus RWM versus RWM in cases of ISR.

CCM	Studies (*n*)	RR (95% CI)	*I* ^2^
decoction	18	0.35 [0.26, 0.46]	0%
granules	2	0.39 [0.13, 1.21]	0%
pills	3	0.59 [0.34, 1.01]	0%
capsules	9	0.49 [0.36, 0.67]	0%
tablets	1		

**Table 7:** Effect of CCM plus RWM versus RWM on Adverse Reactions.

Study	Intervention	EG		CG		RR [95% CI]
		EE	Total	EE	Total	
WANG X 2016	Huoxue yiqi decoction plus RWM versus RWM	8	64	5	64	1.60 [0.55, 4.63]
ZHANG Q 2013	Shenshao Oral Lotion plus RWM versus RWM	2	473	1	477	2.02 [0.18, 22.17]

EG = experimental group; CG = control group; EE = events.

**Table 8:** Effect of CCM plus RWM versus RWM on MACE after PCI.

MACE	No. of studies/patients	Follow-up (mos.)	Pooled estimate (RR, 95% CI)
Revascularization (CCM plus RWM versus RWM)	3/308	6–18	0.33 [0.07, 1.62]
Myocardial Infarction (CCM plus RWM versus RWM)	3/308	6–18	0.33 [0.17, 1.62]
Myocardial Infarction (CCM plus RWM versus CM placebo plus RWM)	2/475	6	0.42 [0.11, 1.60]
Cardiac Mortality (CCM plus RWM versus RWM)	5/921	12–18	0.25 [0.03, 2.21]
Recurrent Angina (CCM plus RWM versus RWM)	9/1095	6–18	0.50 [0.38, 0.65]
Recurrent Angina (CCM plus RWM versus CM placebo plus RWM)	2/475	6	0.32 [0.19, 0.54]

**Table 9:** Effect of CCM on MLD.

Study	Intervention	EG			CG			MD [95% CI]
		Mean	SD	Total	Mean	SD	Total	
DENG XD 2013	Tongxinluo capsule plus RWM versus RWM	3.97	0.59	31	5.58	0.67	30	−1.61 [−1.93, 1.29]
WANG X 2016	Huoxue yiqi tongmai decoction plus RWM vs RWM	2.02	0.22	64	1.6	0.2	64	0.42 [0.35, 0.49]
WANG XD 2010	Tongxinluo capsule plus RWM versus CM placebo plus RWM	2.43	0.62	68	1.16	0.87	64	1.27 [1.01, 1.53]
YANGN TL 2016	Tongxinluo capsule plus RWM versus CM placebo plus RWM	2.81	0.41	172	2.46	0.37	171	0.35 [0.27, 0.43]
ZHANG Q 2013	Shenshao oral lotion plus RWM versus RWM	2.11	0.38	473	2.09	0.32	477	0.02 [−0.02, 0.06]

EG = experimental group; CG = control group; MD = mean difference.

**Table 10:** Effect of CCM on LLL.

Study	Intervention	EG			CG			MD [95% CI]
		Mean	SD	Total	Mean	SD	Total	
LU HW 2014	Tongxinluo capsule plus RWM versus RWM	0.43	0.17	90	0.79	0.24	90	−0.36 [−0.42, 0.30]
YANGN TL 2016	Tongxinluo capsule plus RWM versus CM placebo plus RWM	0.21	0.17	172	0.45	0.24	171	−0.24 [−0.28, 0.20]
ZHANG PF 2014	Xinmai futong decoction plus RWM versus RWM	0.5	0.21	61	0.8	0.4	62	−0.30 [−0.41, 0.19]
ZHANG Q 2013	Shenshao oral lotion plus RWM versus RWM	0.62	0.3	473	0.67	0.32	477	−0.05 [−0.09, 0.01]

EG = experimental group; CG = control group; MD = mean difference.
